# Portal Venous Gas After Trans-arterial Radioembolization in Hepatocellular Carcinoma: A Rare but Critical Imaging Finding

**DOI:** 10.7759/cureus.88107

**Published:** 2025-07-16

**Authors:** Ahmed Ali Aziz, Nosheen Omar, Rehan Shah, Muhammad Ali Aziz, Ijlal Akbar Ali

**Affiliations:** 1 Internal Medicine, Capital Health Regional Medical Center, Trenton, USA; 2 Anatomy, University of Health Sciences, Lahore, PAK; 3 Internal Medicine, Bayonne Medical Center, Bayonne, USA; 4 Internal Medicine, University of Kentucky College of Medicine, Lexington, USA; 5 Digestive Diseases and Nutrition, University of Oklahoma Health Sciences Center, Oklahoma City, USA

**Keywords:** hepatocellular carcinoma (hcc), portal venous gas, post-embolization syndrome, transarterial radioembolization, yttrium-90

## Abstract

Transarterial radioembolization (TARE) is a relatively new treatment option available for unresectable hepatocellular carcinoma (HCC). TARE therapy involves the delivery of radiation directly to the tumor to cause tumor necrosis. Yttrium-90 (Y90) is commonly used as a source of radioembolization in TARE. TARE is very well tolerated and has a low rate of complications. Main complications of TARE for HCC include postembolization syndrome and radiation-induced injury to nearby organs such as the liver, gallbladder, and stomach, causing hepatitis, cholecystitis, or gastric ulceration. A side effect not previously described in TARE literature is portal venous gas after TARE therapy. We present the first ever reported case of portal and variceal venous gas in a 77-year-old male patient who had unresectable HCC and had previously failed chemotherapy. He underwent Y90 TARE for HCC. Following TARE, he presented with right upper quadrant abdominal pain, and imaging showed portal and variceal venous gas. He was treated with antibiotics, with resolution of symptoms and improvement in portal and variceal venous gas on repeat imaging.

## Introduction

Hepatocellular carcinoma (HCC) is the third most common cause of cancer-related mortality worldwide. It is the sixth most common malignancy in the world [1]. The treatment for HCC is based on staging, which considers tumor characteristics, liver function, and overall patient health. The Barcelona Clinic Liver Cancer (BCLC) staging system is a widely used staging system for HCC and categorizes HCC into five stages (0, A, B, C, and D) to guide treatment decisions. Curative treatments like surgical resection or liver transplant are considered for early stages (0 and A), while treatments like TACE or systemic therapy are used for advanced stages (B and C). Patients with end-stage disease (D) receive supportive care and palliation [2].

Transarterial radioembolization (TARE) is a relatively new treatment option for unresectable HCC [3,4]. TARE therapy involves the use of Yttrium-90 (Y90) radiation therapy directed selectively to the hepatic segments with tumor to induce necrosis by occlusion of the vessel feeding the tumor. TARE works on the principle that primary and secondary hepatic tumors are usually vascularized by arterial blood supply, while healthy liver tissue obtains blood supply mostly from the portal network. Hence, TARE has a low side effects profile as therapy is directed directly to the tumor through the arterial supply, and surrounding structures are spared [5]. Common complications include pain, fever, nausea, and vomiting, often termed as postembolization syndrome (PES), along with radiation-induced injury to neighboring organs, causing hepatitis, gastritis, and cholecystitis [6]. Portal and variceal venous gas after TARE therapy is a side effect not previously found in TARE literature, secondary to radiation-induced necrosis of HCC. Here, we present a case of a 77-year-old man with a history of nonresectable HCC and underlying cirrhosis who underwent TARE therapy and presented with right upper quadrant abdominal pain associated with diarrhea. Imaging revealed portal and variceal venous gas. He was started on antibiotics, his symptoms improved, and repeat imaging showed improvement in portal and variceal venous gas.

## Case presentation

**Figure 1:**
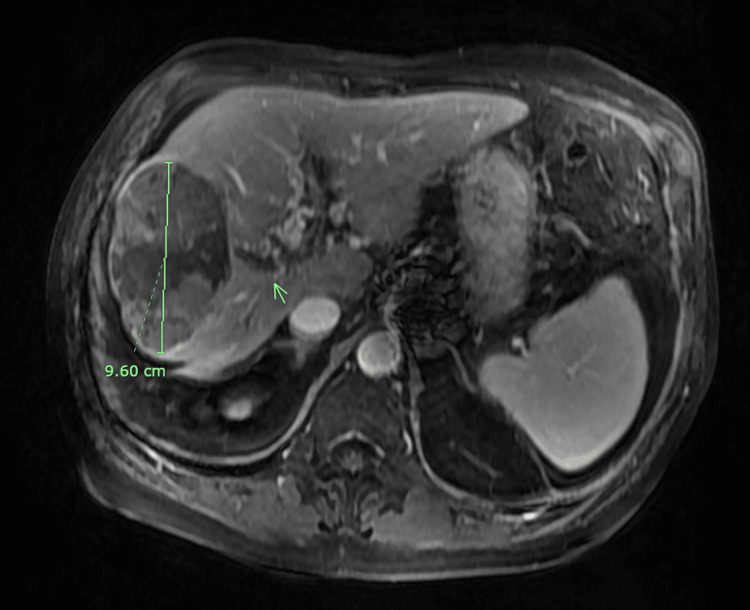
Magnetic resonance imaging of the abdomen with and without contrast transverse section. The green line shows the 9.6 cm hepatocellular carcinoma in the right lobe of the liver. The green arrow points at the hepatocellular carcinoma nodules in the periphery of the large lesion.

A 77-year-old man presented to our emergency department with generalized abdominal pain and multiple episodes of new-onset non-bloody diarrhea ongoing for the past six days. He denied any nausea or vomiting. He had a past medical history of liver cirrhosis secondary to hepatitis C virus, BCLC Stage B infiltrative HCC with thrombosis in the main, right, and left portal veins that was diagnosed 2 - 3 months prior to presentation to our ER, hypertension, and esophageal varices. He had previously received bevacizumab and atezolizumab therapy for treatment of HCC, which were discontinued due to hematochezia. He underwent TARE of segment VIII of the HCC lesion using Y90 radiation eight days ago. His magnetic resonance imaging (MRI) of the abdomen with and without intravenous (IV) contrast, done two months prior to his TARE procedure, showed a 9.6 cm HCC lesion in segment VIII of the liver (Figure 1).

On presentation, his vital signs were stable. His blood pressure was 135/85 mmHg, his pulse was 68 beats per minute, he was afebrile and had a respiratory rate of 18 respirations/minute, and his oxygen saturation was 98% on room air. On physical exam, his abdomen was soft, non-distended, with generalized tenderness to palpation and normal bowel sounds. His blood work was significant for normal complete cell count, normal lactic acid levels, and elevated creatinine to 1.47 mg/dL. Blood cultures and stool testing including Clostridium difficile and gastrointestinal (GI) biofire panel were sent (Table 1).

**Table 1:** Laboratory values at admission. TEM PCR: Target Enriched Multiplex Polymerase Chain Reaction

Lab Parameters (units)	Reference Range	Lab Result
Sodium (mmol/L)	135 – 145	139
Potassium (mmol/L)	3.5 – 5.5	4.2
Chloride (mmol/L)	96 – 106	105
BUN (mg/dL)	6 – 24	16
Creatinine (mg/dL)	0.7 – 1.3	1.47
Bicarbonate (mmol/L)	22 – 29	20
Glucose (mg/dL)	70 - 100	96
Aspartate Aminotransferase (U/L)	13 – 39	36
Alanine Aminotransferase (U/L)	7 – 52	41
Alkaline Phosphatase (U/L)	34 – 104	98
Total Bilirubin (mg/dL)	0.2 – 1.3	0.8
White Blood Cells (k/uL)	3.8 – 10.2	6.2
Hemoglobin (g/dL)	12.9 – 16.7	13.6
Hematocrit (%)	39.2 – 48.8	43.0
Platelets (k/uL)	150 – 450	219
International Normalized Ratio (INR)	0.8 - 1.1	1.6
Partial thromboplastin time (seconds)	25 – 35	28
Prothrombin Time (seconds)	11 – 13.5	17
Lactic Acid (mmol/L)	0.90 – 1.60	1.30
Clostridium difficile Toxin B Gene PCR	Not Detected	Not Detected
Campylobacter jejuni (TEM-PCR)	Not Detected	Not Detected
Cryptosporidium parvum	Not Detected	Not Detected
Enterohemorrhagic Escherichia Coli (EHEC)	Not Detected	Not Detected
Enteroinvasive Escherichia Coli (EIEC)	Not Detected	Not Detected
Enteropathogenic Escherichia Coli (EPEC)	Not Detected	Not Detected
Enterotoxigenic Escherichia Coli (ETEC)	Not Detected	Not Detected
Giardia lamblia	Not Detected	Not Detected
Norovirus	Not Detected	Not Detected
Rotavirus	Not Detected	Not Detected
Salmonella Enterica (TEM-PCR)	Not Detected	Not Detected
Shiga-Like Toxin Gene (STX1)	Not Detected	Not Detected
Shiga-Like Toxin Gene (STX2)	Not Detected	Not Detected
Vibrio Parahaemolyticus (TEM PCR)	Not Detected	Not Detected

**Figure 2:**
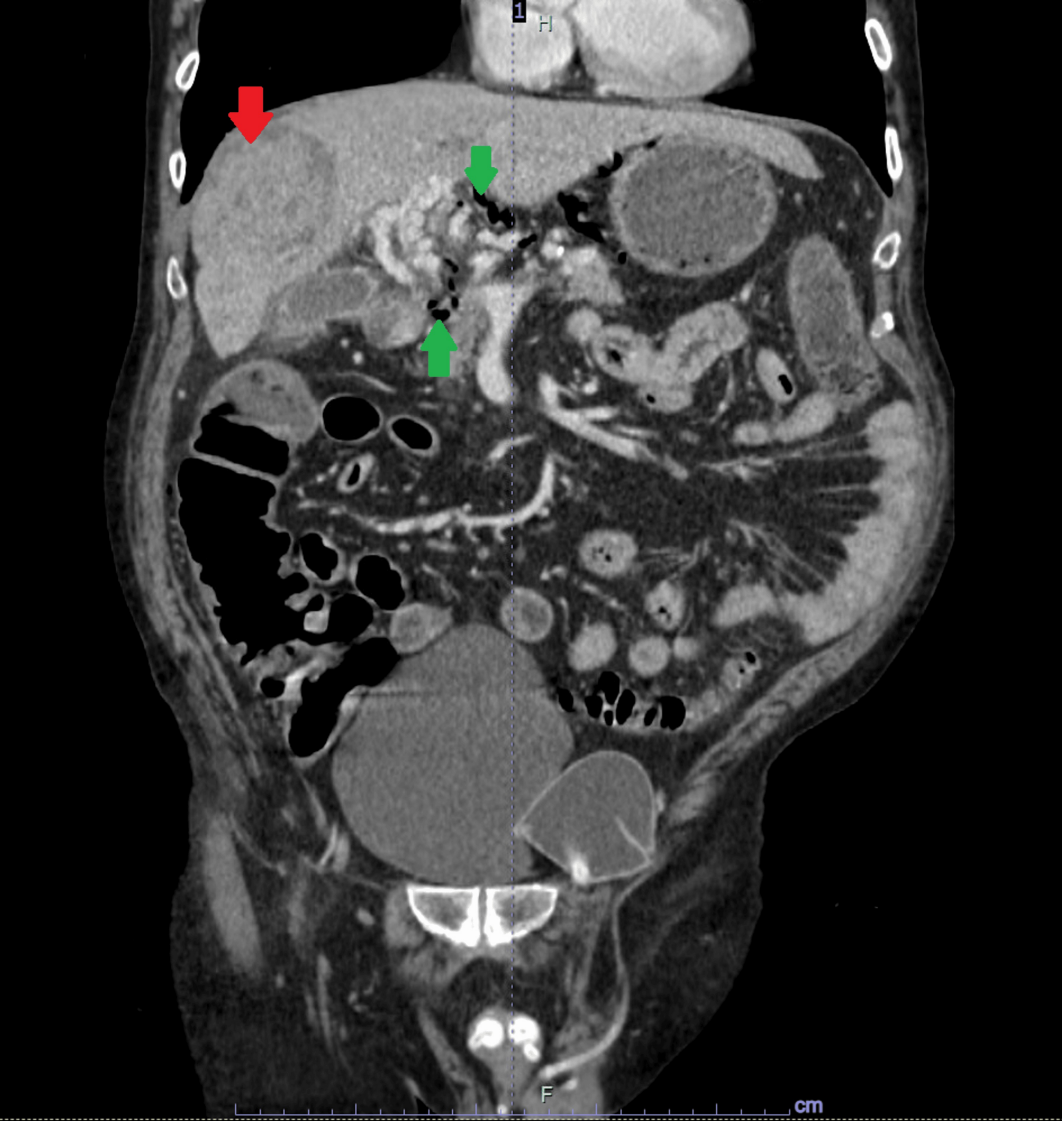
Computed tomography scan of the abdomen and pelvis with intravenous contrast. Portal venous gas is indicated by green arrows. Partially necrotic right hepatic lobe hepatocellular carcinoma treated with Y90 therapy is indicated by a red arrow.

A computed tomography (CT) scan of the abdomen and pelvis (A&P) with IV contrast was done due to abdominal pain. The CT scan showed moderate portal venous gas in the intra-gastric, peri-gastric, and hepatic hilar varices with stable 10 cm partially necrotic mass within the right lobe of the liver compatible with his history of recently treated hepatocellular carcinoma with Y90 therapy (Figure 2).

**Figure 3:**
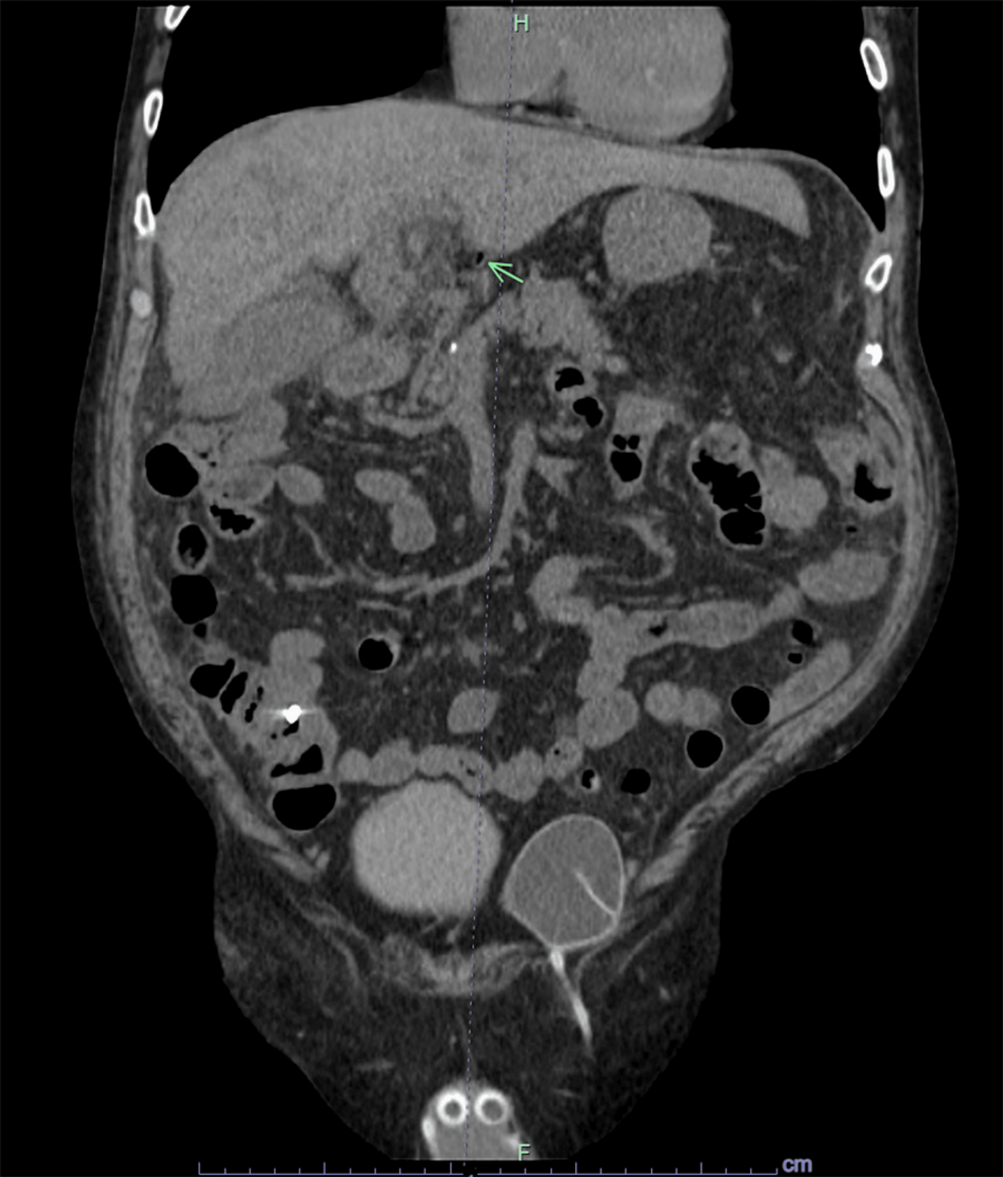
Computed tomography scan of the abdomen and pelvis with intravenous contrast showing improvement in portal venous gas indicated by a green arrow.

The CT A&P did not show any inflammatory conditions of the small or large bowel, emphysematous gastritis, perforated viscus, and obstruction, and the patient denied any retching or vomiting. The portal venous gas was thought to be secondary to recent Y90 radiation therapy to the HCC causing disseminated necrosis in the tumor and production of gas from gas-forming bacteria in necrotic HCC causing dissemination of gas into portal venous system. He was started on antibiotic piperacillin-tazobactam and IV fluids. Hepatology service was consulted and agreed with infectious workup, antibiotics and recommended repeating CT A&P after 48 hours to assess portal venous gas burden. Patient’s diarrhea improved and his clostridium difficile and GI panel were negative. Blood cultures remained negative, and his abdominal pain improved. His creatinine normalized and he was advanced to regular diet. A repeat CT A&P showed marked improvement and reduction in portal venous gas (Figure 3).

He was discharged on oral ciprofloxacin and metronidazole for seven days in a stable condition with outpatient follow-up with his oncologist and hepatologist.

## Discussion

HCC is one of the leading causes of liver-related deaths worldwide [7]. TARE is a new treatment option for unresectable HCC [3,4]. TARE has been shown to increase the time of progression of HCC [8]. Radioembolization with TARE involves the injection of intra-arterial radioactive microspheres like the Y90, which targets the tumor of interest, leading to vessel occlusion and hence causing ischemia and necrosis of the target tumor [9].

TARE therapy generally has good tolerability and a low incidence of complications. This is because pre-procedure angiograms are used to assess vascular distribution to the tumor prior to performing TARE to minimize radioembolization to normal tissues [10]. However, complications still do arise due to the irradiation of non-target tissues, including the tumor-free liver segments [11,12]. The main complications of TARE for HCC are generally classified into PES, GI ulceration, hepatic dysfunction, biliary injury, lymphopenia, and radiation pneumonitis [10]. 

PES is the most common complication after TARE therapy. It presents as clinical symptoms of nausea and vomiting, fatigue, fever, abdominal discomfort, and anorexia, usually within 72 hours of the procedure [13]. Radiation-induced liver damage secondary to TARE therapy is characterized as liver injury in the form of jaundice, liver enzyme derangement, ascites, and elevated alkaline phosphatase [14]. TARE can lead to toxic effects on neighboring organs and cause radiation-induced gastritis, enteritis, or gastroduodenal ulcerations [10]. The clinical course is usually self-limited [15]. Radiation-induced injury to biliary structures might cause gallbladder wall thickening and signs of cholecystitis on imaging. The incidence of cholecystitis post-TARE requiring intervention is low [16]. Migration of Y90 radiation microspheres to pulmonary vasculature from hepatic vasculature can lead to radiation pneumonitis and pulmonary fibrosis [10]. TARE therapy might also cause mild to moderate lymphopenia; however, an increased susceptibility to infections has not been demonstrated [17].

Our patient was a 77-year-old man with a history of nonresectable HCC. He had previously failed treatment with Avastin and Tecentriq. He presented with non-bloody diarrhea and abdominal pain eight days after receiving Y90 TARE therapy. Infectious etiology was ruled out with a negative GI panel, Clostridium difficile testing, and negative blood cultures, which demonstrates the possibility of this being an unusual complication of TARE therapy. His imaging showed portal and variceal venous gas, thought to be secondary to the Y90 radiation therapy to the HCC, causing disseminated necrosis with gas production in the tumor and portal venous system. His clinical condition improved after antibiotic initiation. To our knowledge, this is the first reported case of portal venous gas reported in a patient with HCC after TARE. The TARE procedure is considered to be relatively safe, and patients are usually discharged after the procedure without requiring inpatient monitoring. Our case report is important as it alerts clinicians to be aware of the possibility of late-onset complications such as portal venous gas, like in our patient, which might require hospitalization and treatment with antibiotics.

## Conclusions

TARE is becoming a popular option for the treatment of HCC. It is a relatively safe procedure and has low post-procedure complications, but as with any therapy, knowledge of the potential complications of this therapy is essential. Common complications arise from radiation-induced injury to nearby organs such as the stomach, gallbladder, and liver. TARE is offered as an outpatient treatment, and patients are usually not observed in the hospital. Our case report highlights an important post-procedure complication of TARE, i.e., the development of portal and variceal venous gas, likely from radiation-induced necrosis of HCC. Since our patient presented eight days after his TARE therapy and had necrotic HCC on imaging without inflammatory conditions of the small or large bowel, emphysematous gastritis, perforated viscus, or obstruction on imaging, it was reasonable that necrotic HCC and portal venous gas were from his recent TARE therapy.

Physicians should be aware of late-onset complications of TARE and should educate their patients about these complications. Patients should be asked to seek care in the hospital if they develop abdominal pain and diarrhea post-TARE therapy. Our case report is also important as it adds to the limited literature available on TARE, which is a relatively new treatment option for HCC and is still being explored. Future systemic literature reviews or multicenter research will help elucidate the mechanistic aspects and incidence of such complications.
